# Abundance of Two *Pelagibacter ubique* Bacteriophage Genotypes along a Latitudinal Transect in the North and South Atlantic Oceans

**DOI:** 10.3389/fmicb.2016.01534

**Published:** 2016-09-28

**Authors:** Erin M. Eggleston, Ian Hewson

**Affiliations:** ^1^Department of Microbiology, Cornell UniversityIthaca, NY, USA; ^2^Biology Department, St. Lawrence UniversityCanton, NY, USA

**Keywords:** pelagiphage, phage, *Pelagibacter ubique*, Atlantic ocean, latitude

## Abstract

This study characterizes viral and bacterial dynamics along a latitudinal transect in the Atlantic Ocean from approximately 10 N–40 S. Overall viral abundance decreased with depth, on average there were 1.64 ± 0.71 × 10^7^ virus like particles (VLPs) in surface waters, decreasing to an average of 6.50 ± 2.26 × 10^5^ VLPs in Antarctic Bottom Water. This decrease was highly correlated to bacterial abundance. There are six major water masses in the Southern Tropical Atlantic Ocean, and inclusion of water mass, temperature and salinity variables explained a majority of the variation in total viral abundance. Recent discovery of phages infecting bacteria of the SAR11 clade of *Alphaproteobacteria* (i.e., pelagiphages) leads to intriguing questions about the roles they play in shaping epipelagic communities. Viral-size fraction DNA from epipelagic water was used to quantify the abundance of two pelagiphages, using pelagiphage-specific quantitative PCR primers and probes along the transect. We found that HTVC010P, a member of a podoviridae sub-family, was most abundant in surface waters. Copy numbers ranged from an average of 1.03 ± 2.38 × 10^5^ copies ml^−1^ in surface waters, to 5.79 ± 2.86 × 10^3^ in the deep chlorophyll maximum. HTVC008M, a T4-like myovirus, was present in the deep chlorophyll maximum (5.42 ± 2.8 × 10^3^ copies ml^−1^ on average), although it was not as highly abundant as HTVC010P in surface waters (6.05 ± 3.01 × 10^3^ copies ml^−1^ on average). Interestingly, HTVC008M was only present at a few of the most southern stations, suggesting latitudinal biogeography of SAR11 phages.

## Introduction

Viruses are abundant, diverse, and play a dynamic role in aquatic ecosystems (Breitbart, 2012; Brum and Sullivan, 2015). In marine ecosystems they strongly influence biogeochemical cycling through viral lysis of their hosts. Lysis releases dissolved organic matter and other limiting nutrients which impacts carbon, nitrogen, phosphorous and sulfur cycles (Brussaard et al., 2008; Fuhrman, 2009). Bacteriophages are responsible for host mortality ranging from 10 to 50% per day (Weinbauer, 2004). They also play a critical role in bacterioplankton community structure and are partially implicated in maintaining bacterial diversity (Middelboe et al., 2001; Parada et al., 2008). Recent studies investigate the biogeography of phages, and their hosts, throughout the oceans (Thurber, 2009; Clokie et al., 2011; Chow and Suttle, 2015). While more research is needed, there is clear evidence of spatial and temporal diversity among phages (Marston et al., 2013; Huang et al., 2015). Viral dynamics are contingent upon many biotic and abiotic factors. Viral cycle, lysogenic or lytic, is often controlled by host population density and various studies have shown the impact of temperature, salinity, and oxygen on viral communities (Brum et al., 2013). While we have learned a lot with regard to marine viruses, the dynamics of individual viruses infecting significant components of bacterioplankton communities, especially non-cyanobacterial taxa, is not fully resolved in open ocean plankton.

Bacteriophage-host dynamics have been characterized in the North Atlantic (De Corte et al., 2012) showing that viral to bacterial ratios in the epipelagic zone probably influenced those lower in the water column with thermohaline circulation pattern driving bacterioplankton abundance. The dynamics of bacteriophage and bacterioplankton in the equatorial and southern Atlantic have yet to be characterized in this way. There are six major water masses present in this region of the ocean; the surface waters, defined as approximately 5 m in this study and the deep chlorophyll maximum; the mesopelagic; the Antarctic Intermediate Water (AAIW); the North Atlantic Deep Water (NADW); and the Antarctic Bottom Water (AABW). Many parameters are used to characterize these water masses; the most common include temperature, density, salinity, and organic matter composition (Morozov et al., 2010). Given abiotic differences in these water masses, we anticipate differences in bacterial and viral abundance. Additionally, since bacteriophages often dominate the viral community we would expect a strong correlation between viral and bacterial abundance, as is observed in many environments, with typical bacterial and viral abundance of 10^4^–10^6^ cells ml^−1^ and 10^6^–10^8^ viruses ml^−1^, respectively (Proctor and Fuhrman, 1990; Fuhrman, 1999; Winter et al., 2010; Chow et al., 2014).

SAR11, a clade within the *Alphaproteobacteria*, is an abundant bacterial group in surface waters of oceans around the world (Morris et al., 2002). Fluorescence *in situ* hybridization microscopy of this clade has shown its distribution to be high in coastal and open ocean waters with the greatest relative contribution to bacterial community in the open ocean at high temperatures and low chlorophyll concentration (Lefort and Gasol, 2013). Some research suggests that some SAR11 may contain lysogenic phage (Hewson and Fuhrman, 2007). The ability of SAR11 to resist viral lysis has been debated, with small genome and slow replication or K-strategist selection as possible mechanisms by which SAR11 evades viral attack (Suttle, 2007; Yooseph et al., 2010). However, four viruses of SAR11, referred to as pelagiphage after their host *Candidatus Pelagibacter ubique*, were recently described and appear to be widely distributed in open ocean to coastal environments (Zhao et al., 2013). While the mechanisms of pelagiphage-host interactions remain uncharacterized, many hypotheses (i.e., “killing the winner” in which highly abundant bacterial communities are lysed by viruses, allowing proliferation of less-dominant and more diverse bacterial community members, Thingstad and Lignell, 1997; Winter et al., 2010) could explain these dynamics, and it is suggested that any number of these mechanisms are possible, and are acting on different time scales, and in conjunction with growth rate and nutrient competition (Van Valen, 1973; Thingstad and Lignell, 1997; Våge et al., 2013). High rates of recombination are proposed as the mechanism of rapid evolution in the SAR11 host (Vergin et al., 2007). These recombination events would allow these dominant bacteria to evade attack, even though it comes into contact with infective phage, whereby any gain in selective fitness of host leads to loss of fitness by the phage. However, the question of top-down and/or bottom-up control of host abundance is still widely debated, and has major biogeochemical implications for these dominant populations of phage and SAR11.

**Figure 1:**
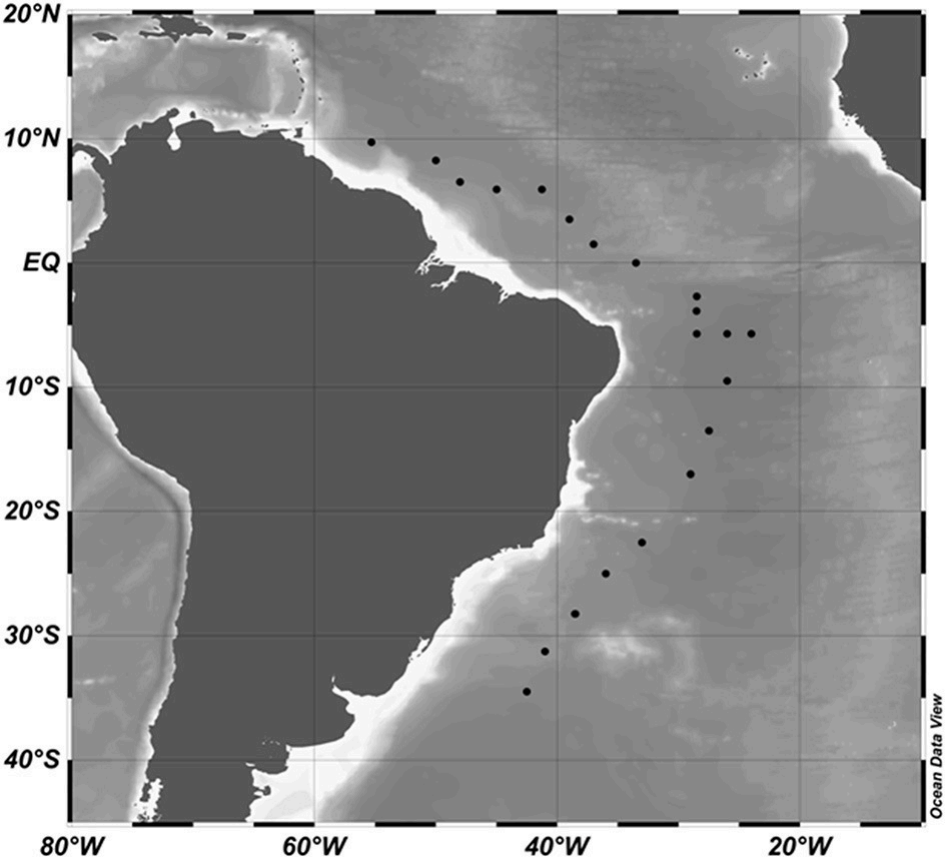
**Cruise track and stations occupied (black dots) during the research cruise KN210-04 between March and May 2013**. Data were plotted in Ocean Data View 4.

This study aimed to quantify, track and examine viral dynamics of two pelagiphage in a latitudinal transect in the Atlantic Ocean. Firstly, we tracked the abundance of two pelagiphage, HTVC010P (a member of a podoviridae sub-family) and HTVC008M (a myovirus) in epipelagic waters along a latitudinal transect from approximately 10 N–40 S in the North and South Atlantic Oceans (Figure 1). Additionally, depth sampling profiles captured the abundance of both bacteria and virus like particles by depth from surface to abyssopelagic waters. Finally, we investigated viral production and pelagiphage dynamics in mesocosm experiments at four latitudes along the transect. We showed that both pelagiphage were present in epipelagic waters, and that HTVC010P was more abundant *in situ* than HTVC008M. However, in the production experiments we only detected HTVC08M. Recent studies provide insight into SAR11 ecotypes and spatiotemporal and ecosystem dynamics (Vergin et al., 2013; Fuhrman et al., 2015; Cram et al., 2016; West et al., 2016). Our data suggest ecotype specific populations of SAR11 phages, as seen in other marine phages (Marston et al., 2013; Chow and Suttle, 2015), and provide initial insight into different viral mechanisms of replication between these two phage.

## Materials and methods

### Sample collection and filtration

Viral samples were collected in the North and South Atlantic from March to May 2013 on cruise 210-04 of the R/V Knorr (Figure 1). Water was collected using a CTD rosette provided by the Knorr Shipboard Science Support Group from six depths: surface waters (approximately 5 m); the DCM designated by peak chlorophyll a concentration (61–160 m); Mesopelagic (250–461 m), AAIW (728–850 m), NADW (2500–2506 m), and the AABW (3492–5526 m); designated by temperature and salinity profiles from the CTD descent at each hydrostation. The CTD system was a SBE9+ CTD (Sea-Bird Scientific, Bellvue, WA, USA) with a depth limit of 6000 m. We used the dual SBE3T/SBE4C sensor system for temperature and conductivity and a SBE43 oxygen sensor (Sea-Bird Scientific). Oxygen data were calibrated based on the discrete water samples analyzed during the cruise using a modified Winkler method (Carpenter, 1965). For each of the six major water depths at each station, approximately 1.5 L of water was sequentially filtered through 25 mm 10 μm Nuclepore and 0.22 μm Durapore membranes (Millipore, Billerica, MA, USA) before capturing on 0.02 μm Anotop-25 filters (Whatman, Pittsburgh, PA, USA). Samples were frozen at −80°C until processed.

### Viral and bacterial abundance

Virus like particles (VLPs) and bacteria (in this paper Bacteria and Archaea), were enumerated by SYBR staining and epifluorescence microscopy (Noble and Fuhrman, 1998; Patel et al., 2007). Briefly, duplicate samples were fixed with 2% formamide (final concentration), filtered over a 25 mm 0.02 μm Anodisc filter (Whatman), stained using SYBR Green 1 dye (Molecular Probes-Invitrogen, Carlsbad, CA, USA) and mounted on slides with a glycerol, PBS, and p-phenylenediamine antifade solution. Slides were stored at −20°C until they were visualized using an Olympus BX51 epifluorescent microscope (Olympus America, Center Valley, PA, USA) to assess viral and bacterial abundance. Approximately 20 VLPs and bacteria were counted in 10 different viewing fields, averaged to the counts per grid box in the viewing field, and then converted to count ml^−1^.

### Viral production experiments

Viral production experiments were carried out to investigate viral production in surface waters (Wilhelm et al., 2002). 2.25 L of surface water (sampled at 5 m depth) was collected by CTD and filtered over a 0.22 μm Durapore (47 mm) membrane filter via vacuum filtration until only 50 mL remained in suspension. A sterile transfer pipette was used to gently resuspend bacteria off of the surface of the filter. This bacterial concentrate (50 mL) was then added to a 2 L, acid washed and seawater-triple rinsed, bottle and filled with 30 kDa tangential flow filtrate (i.e., virus free water; Millipore). Bottles were incubated in a circulating surface water tank with shade cloth to match *in situ* temperature and irradiance. Samples were taken at 0, 12, 24, and 48 h from replicate bottles. At each time point 500 mL were sequentially filtered through a 0.22 μm (25 mm) Durapore membrane filter and 0.02 μm Anotop25 filter for downstream DNA analysis. SYBR slides were prepared from 10 mL of water at each time point to analyze viral and bacterial abundance. Viral decay rate was calculated as the slope of the viral concentration over the time course of the viral production experiment from 0 to 48 h (Noble and Fuhrman, 1997). Host mortality rate estimates were calculated in two steps. Firstly, host requirement (HR) was calculated as the decay rate times VA_0_, and then divided by burst size (both 20 and 100), where VA_0_ was viral abundance ml^−1^ at time zero. Secondly, we estimated the SAR11 population lysed as HR divided by the population of SAR11, assumed to be approximately 50% of the surface water bacteria abundance ml^−1^ (Morris et al., 2002).

### DNA extraction

DNA was extracted from Anotop filters using a modified Zymo Viral DNA Extraction kit (Zymo Research, Irvine, CA, USA). A sterile, flame-sealed, pipette tip was used to stopper the end of the anotop filter that was then filled with 800 μl of ZR Viral DNA buffer. After equilibrating for 10 min, the liquid was removed by syringe and then any remaining buffer was expunged after cracking the filter with a sterile pipette tip. The rest of the protocol followed the manufacturer's instructions except that the DNA was ultimately eluted in nuclease free water.

### *Pelagibacter ubique* bacteriophage primer design

Using the genomes of recently described phage of SAR11 (pelagiphage) HTVC008M and HTVC010P (NCBI Reference sequences NC_020484.1 and NC_020481.1), two viruses were tracked in surface waters and viral production experiments. HTVC008M was chosen as the only myovirus, and HTVC010P was chosen as the most abundant of the three Podiviridae as described (Zhao et al., 2013). While there are no universally conserved genes in viruses, we chose genes that are conserved within certain viral groups: the gene for the major capsid of T4-like myovirus HTVC008M (Hambly et al., 2001), and the head-tail connector gene for HTVC010P (Volozhantsev et al., 2012) for TaqMan primer design with the PRIMER3 program (Rozen and Skaletsky, 2000) as previously described (Short et al., 2010). Briefly, primer design parameters were as follows: minimum, optimum, and maximum length were set as 16, 18, or 20 bp, and minimum, optimum, and maximum melting temperature as 57°, 59.5°, or 63°C. The probe design parameters were set as: minimum, optimum, and maximum length of 22, 25, or 27 nt, and minimum, optimum, and maximum melting temperature of 67°, 70°, or 72°C. BLAST (Altschul et al., 1990) comparison of primers to NCBI's non-redundant database confirmed *in silico* specificity of the primer, probe, and standard sequences. The HTVC008M amplicon was 79 bp, and the HTVC010P amplicon was 87 bp. Table 1 provides details of the primer/probe sets.

**Table 1:** **Pelagiphage qPCR primers, probes, and standards for HTVC008M and HTVC010P**.

**Genotype**	**Sequence (5′-3′)**	Annealing Temperature^*^
08M^‡^_MC-F	GCGGATTCAGATTTCTCTGG	59°C
08M_MC-R	CGGATGTTGTTGTGTCGTTC	
08M_MC-Probe	TGGAACACATTCAGCATCATTGAATCC	
08M_MC-Std	AAGCGGATTCAGATTTCTCTGGTACTGGAACACATTCAGCATCATTGAATCCTGGTTTAATGAACGACACAACAACATCCGTT	
10P^^^_HTC-F	GAAATGCAACAGATGCAACA	55°C
10P_HTC-R	TGCTTCTTCTGGCAATGCT	
10P_HTC-Probe	GCAGGAGGAGATATAGCACCACTAGCG	
10P_HTC-Std	AAGAAATGCAACAGATGCAACAACTACAACAAGTTGCTAAAGCAGGAGGAGATATAGCACCACTAGCGAAAGCATTGCCAGAAGAAGCAAGA	

‡ *HTVC008M*.

^ *HTVC010P*.

* *As determined by gradient standard curves*.

### Quantitative PCR

Real-time quantitative PCR reactions were carried out in triplicate with standards and at least 2 no template controls per run on a StepOne Plus real-time PCR machine (Applied Biosystems, Foster City, CA, USA). The third sample replicate of each sample was spiked with the 10^8^ standard to ensure no amplification inhibition occurred. Each sample reaction (25 μl) contained final concentrations of 1x TaqMan master mix (Applied Biosystems), 10 pmol each of forward and reverse primers and probe, and 0.5 μl template DNA, q.s. nuclease free water. Cycling conditions were as follows: an initial heating step at 50°C for 10 min, followed by a hot start at 95°C for 5 min. Next the mixtures were thermally cycled at 95°C for 30 s followed by 1 min at the appropriate annealing temperature (Table 1), for 50–60 cycles. Cycle threshold was calculated automatically by the instrument's software for calculating gene abundance. *R*^2^ values of the standards for all reactions were greater than 0.97. Gene copy number per reaction was determined by comparison of cycle threshold crossing based on eight standards ranging from 10^4^ to 10^11^ copies per standard reaction.

### Statistical analyses

Summary statistics and regression analyses were performed in the base package of R (R Development Core Team, 2012). Multiple linear regression models were forward selected, only water mass was used as a categorical variable, all others were continuous. The significance of independent variables, adjusted *R*^2^ for the model, and Akaike information criterion values were used to determine the final predictive model. All code and raw data used for these analyses can be found on github (https://github.com/eme47/Pelagiphage).

## Results

### Water column physicochemical variables

Mean temperature across the Atlantic Ocean latitudinal transect, from approximately 10 N–40 S, was greatest in surface waters (27.01 ± 1.80°C), and was lowest in the AABW (1.27 ± 0.70°C). Using the average between the two CTD salinity sensors, mean salinity ranged from 34.47 to 36.46 PSU with the AAIW having the lowest salinity and the DCM having the highest. Mean oxygen concentration was highest in the NADW (5.82 ± 0.12 mL/L) and lowest in the mesopelagic waters (3.62 ± 1.05 mL/L) (Table 2).

**Table 2:** **Mean physicochemical parameters and viral, bacterial and pelagiphage abundance by water mass**.

**Parameter**	**Surface**	**DCM**	**Mesopelagic**	**AAIW**	**NADW**	**AABW**
Temperature (°C)	27.01 ± 1.81	23.81 ± 2.71	12.64 ± 2.48	5.03 ± 0.44	3.03 ± 0.06	1.27 ± 0.70
Salinity (PSU)	36.43 ± 0.93	36.46 ± 0.29	35.26 ± 0.30	34.47 ± 0.10	34.94 ± 0.01	34.77 ± 0.08
Oxygen (mL/L)	4.77 ± 0.14	4.81 ± 0.43	3.62 ± 1.05	3.93 ± 0.83	5.82 ± 0.12	5.36 ± 0.36
Viral Abundance (mL^−1^)	1.17 ± 0.38 × 10^7^	1.64 ± 0.71 × 10^7^	3.17 ± 2.32 × 10^6^	1.57 ± 0.81 × 10^6^	8.24 ± 1.51 × 10^5^	6.50 ± 2.26 × 10^5^
Bacterial Abundance (mL^−1^)	1.52 ± 1.15 × 10^6^	9.31 ± 5.25 × 10^5^	2.20 ± 1.31 × 10^5^	7.73 ± 2.99 × 10^4^	3.56 ± 2.62 × 10^4^	2.45 ± 1.66 × 10^4^
VBR	10.34 ± 4.78	21.50 ± 11.84	15.14 ± 7.81	22.94 ± 15.04	30.27 ± 11.09	29.95 ± 8.46
HTVC008M (copy number mL^−1^)	6.05 ± 3.01 × 10^3^	5.42 ± 2.8 × 10^3^	–	–	–	–
HTVC010P (copy number mL^−1^)	1.03 ± 2.38 × 10^5^	5.79 ± 2.86 × 10^3^	–	–	–	–

*qPCR was only used to detect pelagiphage in Surface and DCM water masses*.

### Viral and bacterial abundance

**Figure 2:**
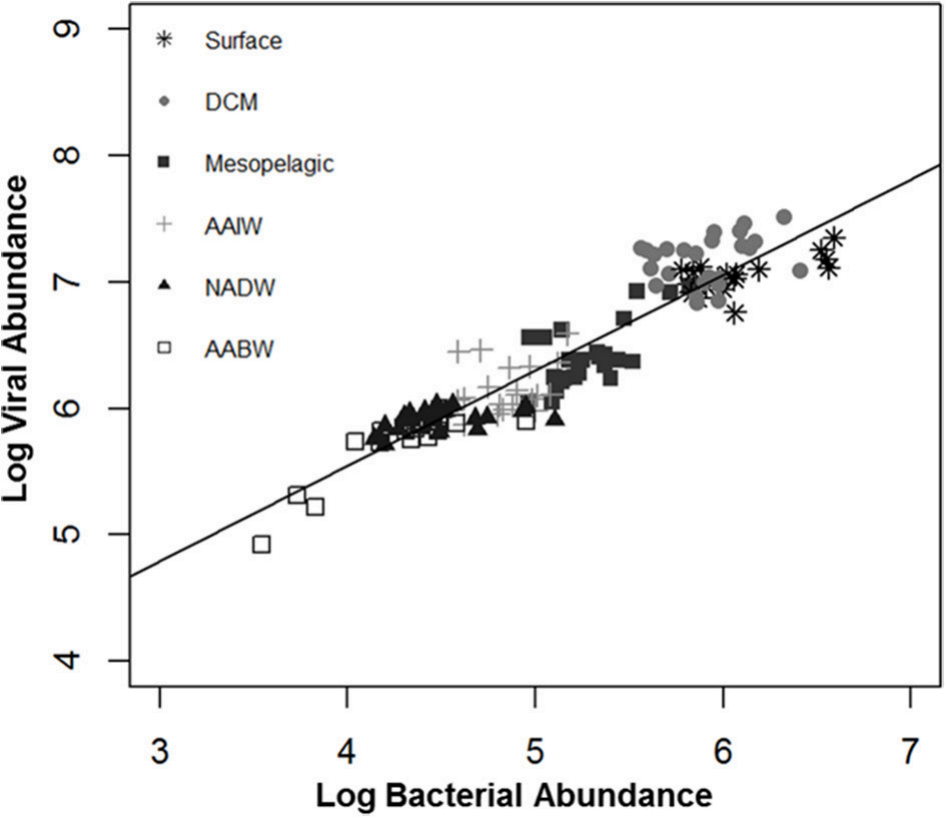
**Relationship between log viral abundance and log bacterial abundance by water mass**. DCM, deep chlorophyll maximum; AAIW, Antarctic intermediate water; NADW, North Atlantic deep water; AABW, Antarctic bottom water.

Mean viral abundance by depth ranged from 6.50 × 10^5^ in AABW to 1.17 × 10^7^ in surface water. Mean bacterial abundance by depth ranged from 2.45 × 10^4^ in AABW to 1.52 × 10^6^ in surface water. Across all depths and stations the viral abundance mean was 5.57 × 10^6^ VLP and mean bacterial abundance was 4.32 × 10^5^ cells. Viral and bacterial abundance were generally highest in surface waters and water from the DCM (Figure 2). Viral to bacteria ratios (VBR) were variable by depth, however an ANOVA of VBR by water mass shows that at least one mean was significantly different from the others (*p* = 0.0259). The NADW and AABW had the highest VBR means (30.27 ± 11.09 and 29.95 ± 8.46, respectively), with the lowest means occurring in the surface and mesopelagic waters (10.34 ± 4.78 and 15.14 ± 7.81, respectively; Table 2). Supplemental Figure 1 shows viral and bacterial abundance, and VBR, by latitude.

A simple linear regression (model 1) indicated that log bacterial abundance (LBA) explains 87.54% (*p* < 0.0001) of the variability in log viral abundance (LVA) (Figure 2). In the multiple linear regression (model 2) water mass, a categorical variable for depth, was also a significant predictor of LVA (Table 3). Mesopelagic, AAIW, NADW, and AABW were negatively associated with LVA when compared to surface waters (*p* < 0.001 for all), while DCM was positively associated with LVA in comparison to surface waters (*p* < 0.001). The inclusion of water mass (i.e., categorical depth) in the model increased percentage LVA variance explained by the model to 91.67% (*p* < 0.0001). Inclusion of temperature and salinity further increased the percentage of variance explained by the model to 91.78% (*p* < 0.0001), however there is no statistical difference between model 2 and model 3. After inclusion of temperature and salinity (model 3), DCM was the only water mass that remained a significant, and positive, predictor of LVA. Salinity was only a nominally significant predictor of LVA while temperature was not significant. The addition of other variables (i.e., oxygen concentration) did increase the predictive power of the model indicating that given the parameters tested we were not able to account for approximately 6% of the variability. The Akaike information criterion (AIC) value was very similar for both models 2 and 3 (−96.4227 and −96.4323, respectively) with a marginal, but not significant, increase in the AIC after the inclusion of temperature and salinity (ΔAIC = −0.0096). This indicates that models 2 and 3 are very similar and provide nearly equal parsimonious fits of the data.

**Table 3:** **Regression analyses with different parameters explaining log viral abundance**.

**Regression Variables**	**Model Number**	**Adjusted *R*^2^**	***p*-value**	**AIC**	**Significant partial slope coefficients**
LBA	1	0.8754	<2.2e-16	−46.0899	Intercept and BLA^***^
LBA, water mass	2	0.9167	<2.2e-16	−96.4227	Intercept, BLA, and six levels of water mass^***^
LBA, water mass, salinity^†^	3	0.9178	<2.2e-16	−96.4323	Intercept, BLA, DCM^***^ Salinity^•^

LBA, log bacterial abundance;

*** p < 0.0001,

• *p < 0.1*.

† *Inclusion of temperature in this model did not significantly improve the prediction of log viral abundance*.

### Pelagiphage abundance in surface and deep chlorophyll maxima waters along a latitudinal transect

**Figure 3:**
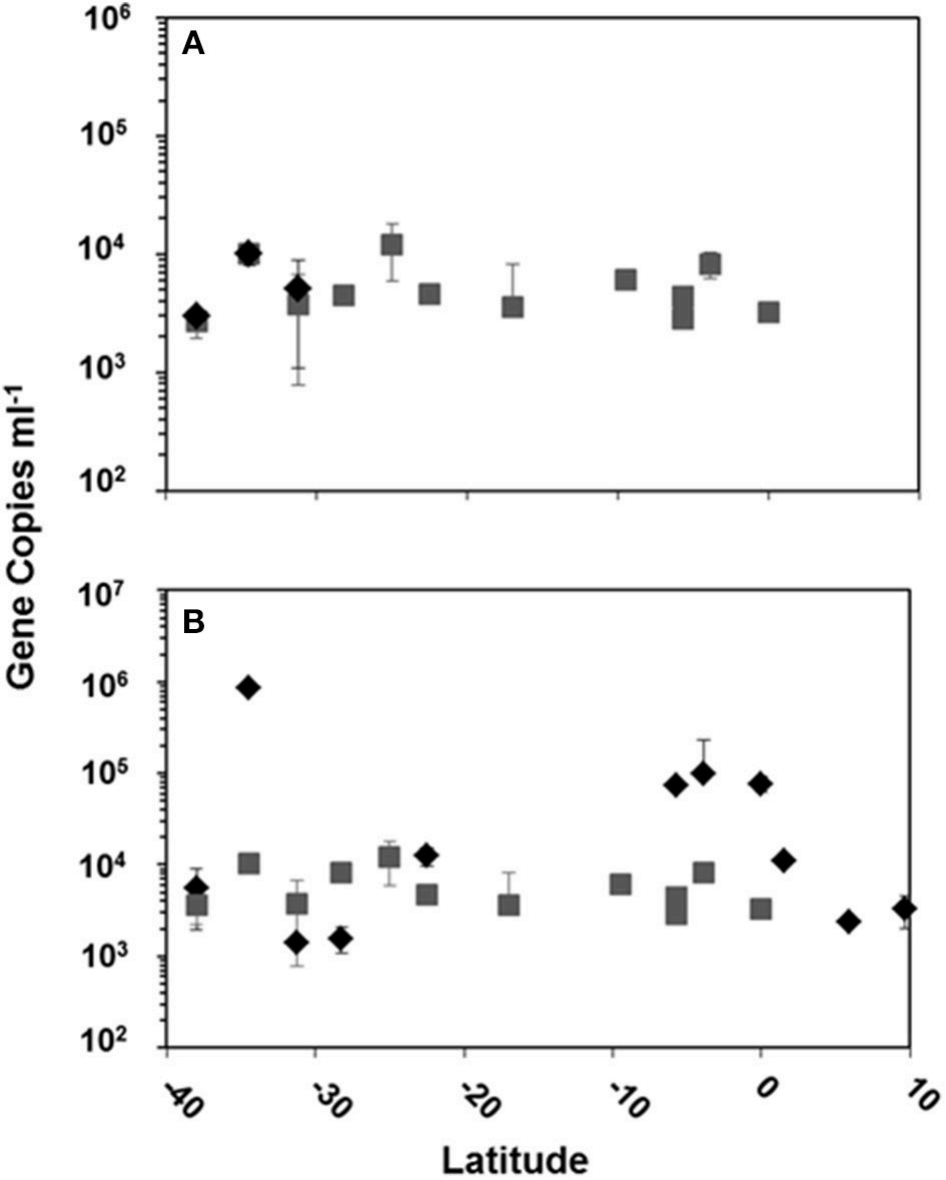
**Copy number in surface water and the DCM of pelagiphage HTVC008M (A) and pelagiphage HTVC010P (B) along the Atlantic Ocean latitudinal transect from 10 N to 40 S**. Water masses are designated as 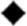 = surface water and 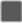 = DCM.

Using quantitative PCR (qPCR) we detected HTVC008M and HTVC010P in DNA extracts only from surface waters and the DCM collected along the latitudinal transect of the cruise, other depths were not sampled in this study. HTVC008M was detected in surface waters at three stations and in the DCM at 13 stations (Figure 3A). HTVC010P was detected in surface waters at 11 stations and in the DCM at the same 13 stations HTVC008M was detected (Figure 3). Surface waters generally had the highest abundance of HTVC010P, although there was strong variation in gene copy number along the transect. Mean HTVC010P copy number in surface water was 1.03 ± 2.38 × 10^5^ copies ml^−1^, with the highest abundance of 8.48 × 10^5^ ml^−1^ (Table 2). HTVC008M was only detected in surface waters at three southern stations in lower abundance relative to HTVC010P. The mean for HTVC008M was 6.05 ± 3.01 × 10^3^ copies ml^−1^. In the DCM HTVC008M and HTVC010P abundance were fairly similar, although they were not detected at every station. HTVC008M mean was 5.42 ± 2.8 × 10^3^ copies ml^−1^ and the HTVC010P mean was 5.79 ± 2.86 × 10^3^ copies ml^−1^. With our limit of detection, the only pelagiphage detected in surface waters of northern latitudes of the transect was HTVC010P.

### Viral abundance and pelagiphage in viral production experiments

**Figure 4:**
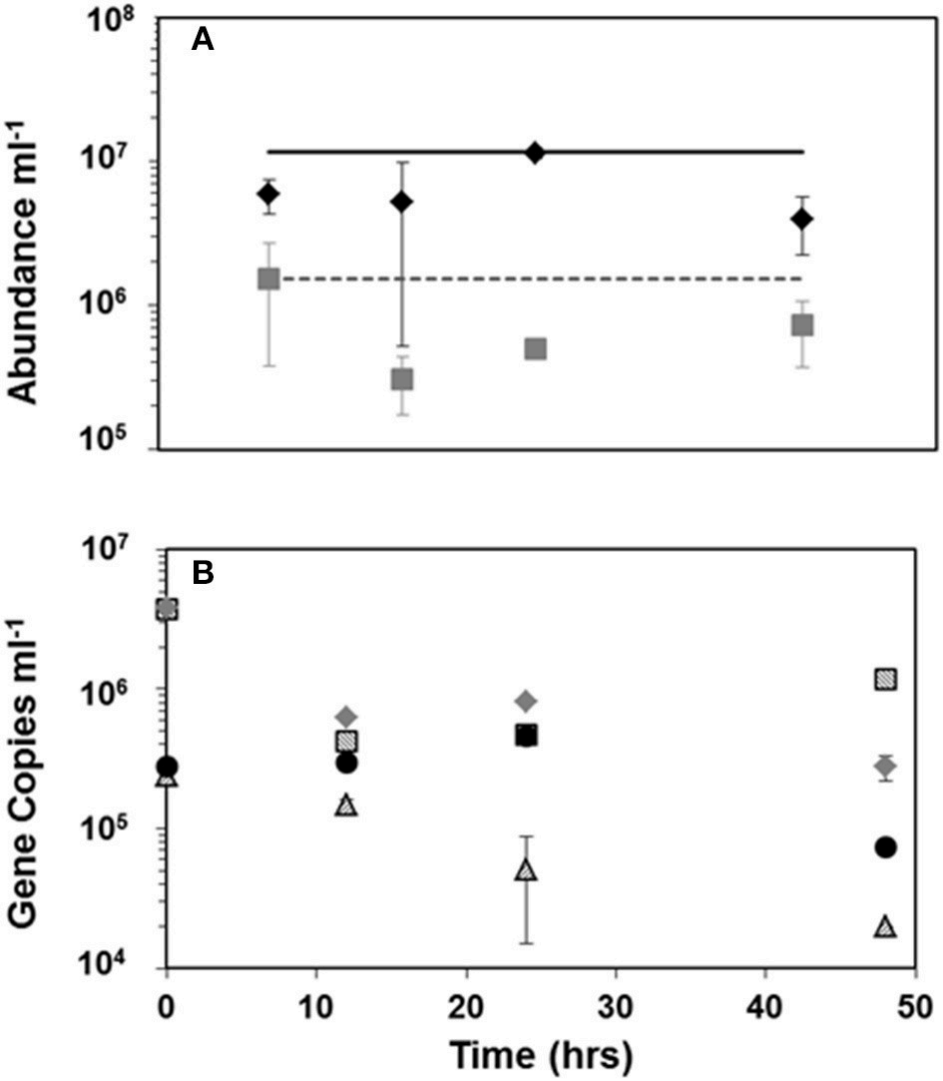
**Mean viral and bacterial counts (A) 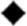 = viral abundance, 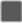 = bacterial abundance, black line is the average ***in situ*** viral abundance and the dashed gray line is the average ***in situ*** bacterial abundance; HTVC008M copy number results (B) from viral production experiments at latitude 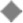 = 38.00S, ■ = 22.49S =, 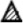 = 2.70S, 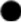 = 9.70N**. Error bars represent standard deviation between means of the four viral production experiments **(A)** or between duplicate qPCR reactions **(B)**.

Viral and bacterial abundance measured at 0, 12, 24, and 48 h of incubation in viral production experiments did not show dramatic changes. Figure 4A shows the averages of four production experiments at each experimental time point. Analysis of mean viral and bacterial abundance at these time points by ANOVA show no significant variation in the means (*p* = 0.463 and *p* = 0.277, respectively).

Tracking pelagiphage in these experiments by qPCR surprisingly revealed high abundance of HTVC008M (Figure 4B) but no HTVC010P within our detection limit. The two production experiments from surface waters with lower latitudes (38.00 and 22.49 S) had higher abundance of HTVC008M than the experiment from a more equatorial latitude (2.70 S) and the one from 9.7 N. At the initial time point there were 3.7 × 10^6^ and 3.8 × 10^6^ copies of HTVC008M per mL from waters collected at stations 38.00 and 22.49 S, respectively. While only two stations, 38.00 and 2.7 S, showed linear decay, we estimate decay as 6.79%/h (*R*^2^ = 0.7955) to 4.99%/h (*R*^2^ = 0.9797), respectively.

## Discussion

### Trends in physicochemical parameters and viral and bacterial abundance across latitude

As expected, salinity and temperature are defining characteristics of the water masses sampled along this transect. Temperature decreased with depth and salinities varied according to water mass (Morozov et al., 2010). Viral abundance ranged from 10^5^ to 10^7^ virus like particles ml^−1^ with lower numbers in deeper waters and bacterial abundance followed a similar pattern with a one log reduction, 10^4^–10^6^ cells ml^−1^. These data match with previous viral and bacterial abundances reported in the open ocean, and that bacterial and viral abundance are negatively correlated with depth (Weinbauer et al., 1995; Wommack and Colwell, 2000; Aristegui et al., 2009). We found that bacterial abundance (model 1, Table 3) accounts for 87.54% of the variability in viral abundance which is much higher than a latitudinal study in the North Atlantic that found bacterial abundance explained 46% of variability in viral abundance (De Corte et al., 2012).

This would suggest that LBA is a better predictor of LVA in this latitudinal transect, however, it does not explain all of the LVA variability. In addition to BLA, water mass, temperature and salinity all increase the predictive power of the model. Water mass accounted for more variation than adding latitude, temperature or salinity, or any permutation of those three variables. This implies that water mass is likely a qualitative indicator of numerous environmental factors, including temperature, salinity, nutrient availability, and other factors. Including BLA, water mass, temperature and salinity explained the highest amount of LVA. The DCM, referent to surface water, remained the only significant water mass in explaining LVA after including temperature and salinity in model 3 (Table 3). This would suggest that there are additional factors in the DCM that relate to viral abundance. Two previous studies revealed that uncoupling between viral and bacterial communities in surface waters was linked to times of high flagellate predation, increased phytoplankton enzymatic activity, and bacterial exoenzymatic activity (Ory et al., 2010, 2011). These authors suggest that bacterial proteolysis contributes to viral assemblage structure (Ory et al., 2011). A study examining epi- and mesopelagic phytoplankton mortality along a North Atlantic transect found that viral lysis was greater at low- and mid-latitudes, with microzooplankton grazing having a greater effect at higher latitudes; this shift was also correlated with temperature, salinity and mixing (Mojica et al., 2016). The DCM harbors diverse eukaryotic life as well, and studies have begun characterizing diatom-bacterial associations (Ghai et al., 2010; Baker and Kemp, 2014). A previous study including found that including picoeukaryotes in their model helped explain viral abundance (Yang et al., 2010). We did not specifically characterize picoeukaryotes, but it is likely that these organisms influence the viral population in the DCM. As mentioned, AIC values for models 2 and 3 were very similar. Therefore, major differences in temperature and salinity are likely characterized by water mass, and the addition of temperature and salinity to the model did not greatly increase the ability to model LVA.

### Pelagiphage in epipelagic waters of the North and South Atlantic Oceans

Pelagiphage HTVC010P, highly represented in Pacific Ocean virome database (Hurwitz and Sullivan, 2013; Zhao et al., 2013), had the highest genotype abundance in surface waters of our study in both the North and South Atlantic. This phage belongs to a subfamily the Podoviridae, and while not much is yet known about Podoviridae in SAR11, they are one of the three major families of dsDNA cyanobacterial phage found ubiquitously in marine environments (Ghai et al., 2010; Wang et al., 2011; Huang et al., 2015). Podoviruses that infect cyanobacteria have a narrow host range and lack known genes for lysogeny which makes them obligately lytic phage (Paul and Sullivan, 2005). More research is needed to determine the reproductive strategies of both HTVC010P and HTVC008M. Both the HTVC010P and HTVC008M pelagiphage had similar genotype abundance in the DCM. HTVC008M genetically clusters with the T4-like myoviruses. Strikingly, HTVC008M was only detected in three southern stations in surface waters. While this pelagiphage was not nearly as abundant relative to HTVC010P in the initial study, it is surprising that it was only present at a few stations. Recent studies mapping the biogeography of different SAR11 ecotypes show unique global distribution of bacteria belonging to this clade (Field et al., 1997; Vergin et al., 2007; Brown et al., 2014; Salter et al., 2015). Differences in infection resistance by some SAR11 ecotypes, as well as temporal differences in their distribution may help elucidate pelagiphage distribution (Fuhrman et al., 2015; Cram et al., 2016). Further research into the SAR11 ecotypes present along this latitudinal transect may help explain the dynamics of pelagiphage location and abundance.

### Viral and pelagiphage abundance and dynamics in viral production experiments

Viral abundance in four viral production experiments remained constant over the time course, however bacterial abundance dropped approximately 10-fold from the initial time point to 12 h and remained lower than the initial abundance until the end point. It is likely that bacterial abundance decrease is linked to bottle effect, as we did not see a major increase in viral abundance that would be expected if lysogenic phage were induced. Previous work with the marine myophage K139 genome shows genes for lysogeny, unlike other characterized marine myoviruses (Kapfhammer et al., 2002; Paul and Sullivan, 2005). Results of the qPCR for the T4-like myovirus HTVC008M showed high abundance in the two viral production experiments at southern latitudes. The viral production experiment from the station at 38 S was one of the only stations where HTVC008M was detected from the latitudinal transect (Figures 3A, 4B). Ambient abundance of HTVC008M at the two Southern stations was lower than the abundance at time zero of the production experiment which would suggest lysogenic induction due to handling. Additionally, although HTVC008M was undetectable at stations further north in the latitudinal survey, the viral production experiments 28.49 S, 2.90 S, and 9.70 N show detectable, albeit not as highly abundant, HTVC008M presence. This suggests that HTVC008M may be a latent infection until induced in these production experiments. HTVC008M integrated in their host would not have been detected as the bacterial size fraction was removed before capturing the viral size fraction prior to DNA extraction. While the genome of HTVC008M does not contain known genes for lysogeny, many of its genes are characterized as hypothetical and their function requires further investigation. Unexpectedly, in all four viral production experiments HTVC010P was undetectable. Given that it was the most highly abundant pelagiphage in surface waters along the latitudinal transect we would have expected to see a high abundance of the phage in these experiments as well. The experimental setup filtered out initial viruses, therefore the lack of HTVC010P at time zero or later time points suggests two possible characteristics about their host: the host growth rate is slow (Rappé et al., 2002); and the host may be quite sensitive to incubation conditions and died. Using the decay rates from latitudes 38.00 and 2.70 S, we estimate a mortality rate for SAR11 host. Assuming SAR11 make up approximately 50% of the total bacterioplankton population (Morris et al., 2002), the mortality rate would range from 0.02 to 1.2% lysed h^−1^ for a burst size ranging from 20 to 100 (Supplemental Table 1).

These data provide insight into the nature of the relationship between viruses and bacteria along a large latitudinal transect, 10 N–40 S, in the North and South Atlantic Oceans. Variation in viral abundance was well characterized by bacterial abundance and water mass, suggesting that host, as well as abiotic factors in these major water masses, shape the viral populations therein. Additionally, we have shown that two pelagiphage, HTVC008M and HTVC010P, have broad distribution in epipelagic waters along this transect. Variation in abundance between these pelagiphage, and between surface waters and DCM, suggest that there are differences in host populations and viral cycles latitudinally. While our data suggest that HTVC008M, similar to cyanomyovirus, may be lysogenic, and HTVC010P, similar to podoviruses of cyanobacteria, is lytic, more research is need to elucidate this dynamic and the mechanism of action and replication of these phage. The distribution of these two pelagiphage hint at latitudinal variation of SAR11 host, and investigation of SAR11 ecotypes in the epipelagic waters will bring clarity to this dynamic. Our data indicate a model in which HTVC008M infect a highly abundant or fast growing and sensitive host. Once released from the host, it decays rapidly and thus we do not detect it as readily in surface waters. Conversely, HTVC010P grows on a slow-growing SAR-11 ecotype, also killed when handled, and decays more slowly upon release in virioplankton. Further investigation of the dynamics of these phage and measurements of decay will help elucidate the role pelgiphage play in shaping host populations. Additionally, characterizing the viruses of other organisms and their viruses in the DCM, and other water masses, may help explain more of the variability we detect in viral abundance.

## Author contributions

EM wrote the article, performed the research, and carried out the analyses. IH provided significant feedback on the manuscript, and funded the research.

### Conflict of interest statement

The authors declare that the research was conducted in the absence of any commercial or financial relationships that could be construed as a potential conflict of interest.

## Supplementary material

Supplementary Figure 1 **Viral abundance (A), bacterial abundance (B) and VBR (C) by latitude**.

Supplementary Table 1 **Estimated Mortality Rate**. ^*^Assumed approximately 50% of surface bacterioplanton population is SAR11 (Morris et al., 2002).
