# Supplementary figures and images for: Abundance of Two Pelagibacter ubique Bacteriophage Genotypes along a Latitudinal Transect in the North and South Atlantic Oceans
Source: Front Microbiol. 2016 Sep 28;7:1534. doi: 10.3389/fmicb.2016.01534 (PMC5039313; doi:10.3389/fmicb.2016.01534)

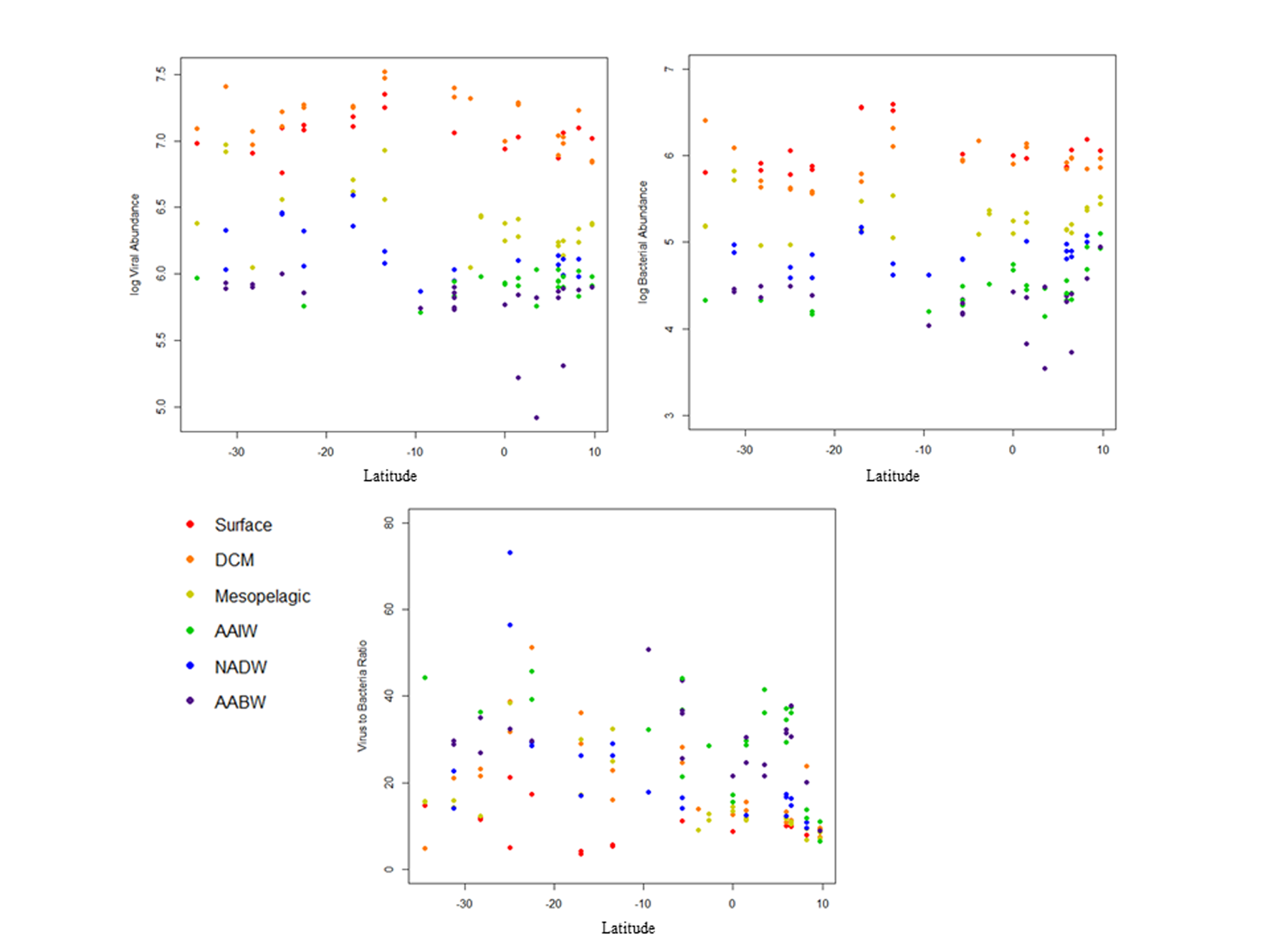

Supplement: Supplementary Figure 1 — Viral abundance (A), bacterial abundance (B) and VBR (C) by latitude. [file Image1.TIF]
