# Supplementary material for: Abundance of Two Pelagibacter ubique Bacteriophage Genotypes along a Latitudinal Transect in the North and South Atlantic Oceans
Source: Front Microbiol. 2016 Sep 28;7:1534. doi: 10.3389/fmicb.2016.01534 (PMC5039313; doi:10.3389/fmicb.2016.01534)
Supplement: Supplementary Table 1 — Estimated Mortality Rate. *Assumed approximately 50% of surface bacterioplanton population is SAR11 (Morris et al., 2002). [file Table1.DOCX]

| Supplemental Table 1: Estimated Mortality Rate | |  |
| --- | --- | --- |
| Lattitude | 38.00 S | 2.70 S |
| Decay Rate | 0.0679 | 0.0499 |
| SAR11 Population* | 7.60E+05 | 7.60E+05 |
| % SAR lysed h^-1^ (burst size=20) | 0.11 | 1.24 |
| % SAR lysed h^-1^ (burst size=100) | 0.02 | 0.25 |
| *Assumed ~50% of surface bacterioplanton population is SAR11 (Morris et al. 2002) | | |
